# Telerehabilitation for People With Physical Disabilities and Movement Impairment: A Survey of United Kingdom Practitioners

**DOI:** 10.2196/30516

**Published:** 2022-01-03

**Authors:** Sarah A Buckingham, Krithika Anil, Sara Demain, Hilary Gunn, Ray B Jones, Bridie Kent, Angela Logan, Jonathan Marsden, E Diane Playford, Jennifer Freeman

**Affiliations:** 1 School of Health Professions University of Plymouth Plymouth United Kingdom; 2 School of Health Sciences University of Southampton Southampton United Kingdom; 3 Centre for Health Technology University of Plymouth Plymouth United Kingdom; 4 School of Nursing and Midwifery University of Plymouth Plymouth United Kingdom; 5 Stroke Rehabilitation Royal Devon and Exeter NHS Foundation Trust Exeter United Kingdom; 6 Warwick Medical School University of Warwick Warwick United Kingdom; 7 Central England Rehabilitation Unit Royal Leamington Spa Rehabilitation Hospital Warwick United Kingdom

**Keywords:** telerehabilitation, physical disabilities, movement impairment, remote assessments, telehealth, rehabilitation, training, health care practitioners, physiotherapy, occupational therapy

## Abstract

**Background:**

Telerehabilitation is a feasible and potentially effective alternative to face-to-face rehabilitation. However, specific guidance, training, and support for practitioners who undertake remote assessments in people with physical disabilities and movement impairment are limited.

**Objective:**

The aims of this survey of United Kingdom–based health and social care practitioners were to explore experiences, assess training needs, and collate ideas on best practices in telerehabilitation for physical disabilities and movement impairment. The aim will be to use the findings to inform a practical tool kit and training package for telerehabilitation use.

**Methods:**

UK rehabilitation practitioners were invited to complete an online questionnaire from November to December 2020. Opportunity and snowball sampling were used to recruit participants from professional and educational networks, special interest groups, and via social media. Closed questionnaire items were analyzed using descriptive statistics. Qualitative inductive analysis using NVivo was used for open responses.

**Results:**

There were 247 respondents, of which 177 (72%) were physiotherapists and occupational therapists. Most (n=207, 84%) had used video-based consultations (typically supported by telephone and email), and the use of this method had increased in frequency since the COVID-19 pandemic. Practitioners perceived telerehabilitation positively overall and recognized benefits for patients including a reduced infection risk, convenience and flexibility, and reduced travel and fatigue. Common obstacles were technology related (eg, internet connection), practical (eg, difficulty positioning the camera), patient related (eg, health status), practitioner related (eg, lack of technical skills), and organizational (eg, lack of access to technology). Support from family members or carers was a major facilitator for successful remote consultations. Of the 207 respondents who had used video-based consultations, 103 (50%) had assessed physical impairments using this method, 107 (52%) had assessed physical function, and 121 (59%) had used patient-reported outcome measures. Although practitioners generally felt confident in delivering video-based consultations, they felt less proficient in undertaking remote physical assessments, expressing concerns about validity, reliability, and safety. Only 46 of the 247 (19%) respondents had received any training in telerehabilitation or video consultations, and some felt they were “feeling their way in the dark.” Practitioners desired training and guidance on physical assessment tools suitable for remote use, when to use video-based consultations or alternative methods, governance issues, digital platforms, and signposting to digital skills training for themselves and their patients.

**Conclusions:**

In response to the COVID-19 pandemic, practitioners rapidly adopted telerehabilitation for people with physical disabilities and movement impairment. However, there are technical, practical, and organizational obstacles to overcome, and a clear need for improved guidance and training in remote physical assessments. The findings of this survey will inform the development of a tool kit of resources and a training package for the current and future workforce in telerehabilitation.

## Introduction

Physical disabilities and impairments are common; globally, one in three people will experience an illness, injury, or impairment that will benefit from rehabilitation at some point in their life [1]. According to the International Classification of Functioning, Disability and Health, impairment is a problem in body function or structure (eg, weakness, tremor, loss of range, or muscle length), which may result in disability (ie, impact on function at an individual or societal level) [2].

Usually, hands-on detailed movement assessment is carried out by practitioners such as physiotherapists, occupational therapists, speech and language therapists, and podiatrists. During the COVID-19 pandemic, disruption of health care services and shielding of the most vulnerable meant that many people did not receive any face-to-face rehabilitation [3,4].

In response, practitioners adapted their practices to incorporate new ways of working, including telerehabilitation—the delivery of rehabilitation services via infosssrmation and communication technologies [5]. The pandemic generated a rapid increase in the use of telephone and video-based consultations for rehabilitation assessments and interventions, in the United Kingdom and worldwide [4,6,7]. Although efficacy is not yet established, systematic review evidence suggests that services delivered using these methods may be as effective as face-to-face interventions for improving patient outcomes [8,9]. In one review, physiotherapy delivered via video or telephone for a range of musculoskeletal conditions was associated with similar or superior improvements in physical function and pain outcomes when compared to usual (face-to-face) care [8]. Another review reported comparable improvements in health-related quality of life of patients with stroke (and their caregivers) in telerehabilitation and control groups [9].

Telerehabilitation is perceived as acceptable by many patients with physical disabilities, including those with chronic musculoskeletal conditions [10], stroke [11], and severe expressive communication disorders [12]. Patient surveys have found that services delivered remotely may be preferred due to advantages such as reduced travel time and convenience [10,13,14]. In addition, there are potential cost savings for health and social care providers when rehabilitation services are delivered remotely; this includes reduced costs associated with practitioners’ time and patients’ and practitioners’ travel [15] in addition to lower outpatient resource use [16].

Our recent global scoping review found that specific published guidance, training, and support on how to undertake remote assessments in people with physical disabilities is limited [17]. Professional bodies and clinical networks highlight large variations in the approaches taken, expressing concerns about potential inequity and inefficiency [4,18,19]. There is a clear need for standardized guidance, support, and training in telerehabilitation for physical disabilities and movement impairment.

To produce guidance that is useful, relevant, and applicable to real-world practice, the experiences and needs of health and social care practitioners must first be understood. As part of a National Health Service (NHS) UK Research and Innovation–Medical Research Council–funded project that aims to develop a tool kit of resources and a training package to support practitioners in carrying out remote physical assessments, we conducted a survey of rehabilitation practitioners.

The objectives of the survey were to:

- Understand UK practitioners’ experiences of telerehabilitation for people with physical disabilities and movement impairment (including use, perceived benefits and obstacles, and outcomes assessed)
- Explore practitioners’ self-perceived confidence and competence in carrying out remote physical assessments
- Identify knowledge gaps and training needs
- Collate examples of best practice and recommendations

## Methods

### Overview of Survey

A cross-sectional online survey was conducted in November and December 2020 using the Jisc platform [20]. Ethical approval was obtained from the University of Plymouth Faculty of Health Staff Research Ethics and Integrity Committee (ref 2392). CHERRIES (Checklist for Reporting Results of Internet E-Surveys) [21] was used to guide the design, conduct, and reporting of the survey.

### Design and Development

Findings from our scoping review [17] and consultation with experts informed the survey questions. The expert consultation process involved informal discussions (email and verbal) with specialists in rehabilitation and physical disabilities, including health and social care practitioners and academics within, and external to, the project team. This enabled the identification of key issues and relevant questions, with a focus on what would practically inform the tool kit and training package.

The questionnaire included a combination of closed response (tick box, multiple choice, and Likert rating scales) and open response (free text) questions. To maximize accuracy and completeness of data, validation and compulsory items [20] were used in the questionnaire design. Only the closed response questions were compulsory and included *prefer not to say*, *other*, and *none of the above* options where appropriate. Adaptive questioning (ie, routing) was also used to ensure that only questions relevant to each respondent were answered [20,21]. Respondents were unable to submit responses until all relevant sections had been worked through and were able to amend their answers during completion. Prior to dissemination, the questionnaire was piloted with members of the research team for usability and technical functionality, with minor changes made to the structure, wording, and order as a result.

The questionnaire consisted of five sections: demographics, experience of telerehabilitation, perceived competence, knowledge and training, and final comments and (optional) contact details. There were 37 questions in total, with additional subquestions. The questionnaire took approximately 15 minutes to complete. A copy of the questionnaire is included in Multimedia Appendix 1.

### Recruitment and Data Collection

Health and social care practitioners involved in rehabilitation throughout the United Kingdom were invited to take part in the survey. A combination of opportunity and snowball sampling were used; potential participants were identified from contacts and networks of the research team, and these participants were in turn asked to forward the survey to other potential participants. Invitations were sent via email to national networks (eg, Therapists in Multiple Sclerosis National Network), professional bodies (eg, Royal College of Occupational Therapists), regional education networks (eg, First Contact Practitioners), and special interest groups (eg, South West Physiotherapy Respiratory Interest Group). The survey was also advertised via social media (Twitter and Facebook). Inclusion criteria were broad; UK-based practitioners involved in rehabilitation were eligible to participate, regardless of their level of experience with telerehabilitation. This included professionals with direct patient contact, who were working in the NHS, social services, independent private, or charitable organization sectors.

The survey was open to anyone with the web link meeting the inclusion criteria. Potential respondents were provided with information on participation, ethical considerations, and use of their data at the beginning of the questionnaire. This was followed by an online consent form. Respondents were informed that their responses would be anonymized for reporting and analysis but were given the option to leave their name and contact details for clarification or discussion of their answers with the research team or to receive future study updates. No incentives were offered for participation.

### Data Analysis

Data cleaning was performed prior to analysis. This included checking the overall data set for duplicate entries and checking individual responses for eligibility. Based on these checks, one respondent based outside of the United Kingdom was excluded.

Quantitative analysis was performed using Excel (Microsoft Corporation) and SPSS Statistics 25 (IBM Corp) [22]. Descriptive statistics (eg, frequencies, percentages, and mean) were calculated for the closed questionnaire responses.

Qualitative thematic analysis was used to analyze the open responses, following the guidance of Braun and Clarke [23] and Braun et al [24]. Qualitative responses were coded and organized using NVivo 12 (QSR International) [25]. Following familiarization with the data, two researchers (authors SAB and KA) independently coded the responses before meeting to compare and discuss the identified themes. Common themes within the data were identified inductively (ie, generated from the data as opposed to guided by theory). Responses to the following questions were analyzed thematically: reasons for not using video-based consultations, concerns regarding validity and reliability of remote physical assessments, ways of overcoming challenges, recommendations for carrying out telerehabilitation with people with physical disabilities and movement impairment, recommendations for video-based consultations with people recovering from COVID-19, open responses on information and training needs, and further comments on telerehabilitation.

## Results

### Demographics of Respondents

In total, 247 health and social care practitioners participated in the survey. Respondents had a mean age of 44.1 (SD 9.8) years, with an age range of 23-70 years. The majority (n=202, 82%) were female. The respondents’ occupational characteristics are shown in Table 1. Almost half (n=114, 46%) of the respondents were physiotherapists, but a large range of allied health and social care professionals were represented. Respondents were from a range of specialties (most frequently neurological and musculoskeletal) and worked in various settings, with the highest proportions working in community health or social care and secondary care.

**Table 1:** Occupational characteristics of survey respondents.

Variable			Respondents (N=247), n (%)^a^
**Profession**			
	Physiotherapist	114 (46)	
	Occupational therapist	63 (26)	
	Prosthetist or orthotist	17 (7)	
	Medic	15 (6)	
	Speech and language therapist	12 (5)	
	Podiatrist	8 (3)	
	Nurse	4 (2)	
	Social worker	3 (1)	
	Dietician	2 (1)	
	Other	9 (4)	
**Setting of service**			
	Community health or social care	91 (37)	
	Secondary care (eg, hospital outpatients)	81 (33)	
	Tertiary care (eg, specialist hospitals)	33 (13)	
	Private practice	14 (6)	
	Primary care (eg, GP^b^ surgeries)	11 (5)	
	Charity or social enterprise	10 (4)	
	Academic institution	4 (2)	
	Residential social care	2 (1)	
	Other	1 (0.4)	
**Clinical specialty**			
	Neurological (including stroke)	103 (42)	
	Musculoskeletal/heumatology	28 (11)	
	Pediatrics	23 (9)	
	Community rehabilitation	20 (8)	
	Care of older people	11 (5)	
	Trauma/orthopedics	12 (5)	
	Developmental/learning	9 (4)	
	Disabilities	8 (3)	
	Amputees	7 (3)	
	Generic	3 (1)	
	Hand therapy	2 (1)	
	Mental health	2 (1)	
	Sports and exercise	2 (1)	
	Vocational services	17 (7)	
	Other or multiple specialties	1 (0.4)	
**Work mainly with...**			
	Adults	186 (75)	
	Children/adolescents	61 (25)	
**Location**			
	England	205 (83)	
	Scotland	22 (9)	
	Wales	16 (7)	
	Northern Ireland	3 (1)	
	Other British isles	1 (0.4)	
**Deliver service predominately to patients/service users in...**			
	Urban setting	106 (43)	
	Rural setting	41 (17)	
	Both	100 (41)	

^a^Percentages may not total 100 due to rounding.

^b^GP: general practitioner.

### Experiences of Telerehabilitation

#### Use of Telerehabilitation

Of the 247 respondents, 207 (84%) reported having used video-based consultations. Respondents recalled their use of video-based consultations before, during, and after the first COVID-19 lockdown in the United Kingdom; the government restrictions imposed between March and June 2020 included 2-meter social distancing rules, restrictions on travel (only essential travel was permitted), and closure of nonessential retail and public venues. The frequency of use had increased substantially during this time; before March 2020, only 27 of 207 (13%) respondents were using video-based consultations, compared with 195 of 207 (94%) respondents after the first COVID-19 lockdown. Video-based methods were typically supported by telephone and email. Practitioners used video-based consultations for a range of purposes including screening and triage, assessments, and intervention delivery (Table 2). The follow-up assessment was the most commonly cited reason for using telerehabilitation, reported by 177 of 207 (86%) respondents. Only 29 of 207 (14%) respondents had delivered virtual group interventions (eg, exercise or educational classes), compared with 129 of 207 (62%) respondents who had delivered individual interventions.

The most frequently used platforms for video-based consultations were Attend Anywhere (Chris Ryan) [26] (used by 124/207, 60% of respondents), Teams (Microsoft Corporation) [27] (used by 79/207, 38%), and Zoom (Zoom Video Communications) [28] (used by 58/207, 28%). More than half of the respondents (112/207, 54%) reported using more than one platform. Organizational requirements were the largest influencing factor in selecting a particular platform, with 178 of 207 (86%) respondents providing this as a reason for their choice. Some practitioners noted a disparity between organizational requirements and which platforms might be preferred by patients:

I am limited by what our organisation considers to be secure which is not what patients are more familiar with.

Occupational Therapist, Neurology

**Table 2:** Purposes of video-based consultations (n=207).^a^

Purpose of consultation	Respondents, n (%)^b^
Screening and triage	80 (39)
Initial assessment	154 (74)
Follow-up assessments	177 (86)
Assess or review use of equipment	77 (37)
Intervention delivery on an individual basis (eg, goal-setting, exercise, or education)	129 (62)
Intervention delivery on a group basis (eg, exercise class or educational class)	29 (14)

^a^Respondents were asked “For which of the following purposes have you used video-based consultations?”

^b^Some respondents used video consultations for multiple purposes; therefore, the percentages do not total 100.

#### Perceived Benefits and Obstacles

Overall, respondents perceived telerehabilitation in a positive light and saw it as a valuable tool and a useful adjunct to, rather than a replacement for, face-to-face care:

We've been talking about telerehabilitation for so long and COVID has made us step up to the plate. Although it is useful, it can never replace face-to-face consultations.

Physiotherapist, Stroke Rehabilitation

[Telerehabilitation is] a useful tool, but in my practice the gold standard is still face-to-face consultation, and is liable to remain so for the foreseeable future. Much of my work involves having to touch, manipulate or adjust prostheses and this cannot be done remotely.

Prosthetist, Amputees

It was recognized that telerehabilitation may not be the best option for every person or case. Examples given where practitioners felt remote consultations were less appropriate were consultations with older people; people with severe cognitive, sensory, or physical impairments; and cases where manual therapy such as adjustment of prostheses is required.

**Figure 1:**
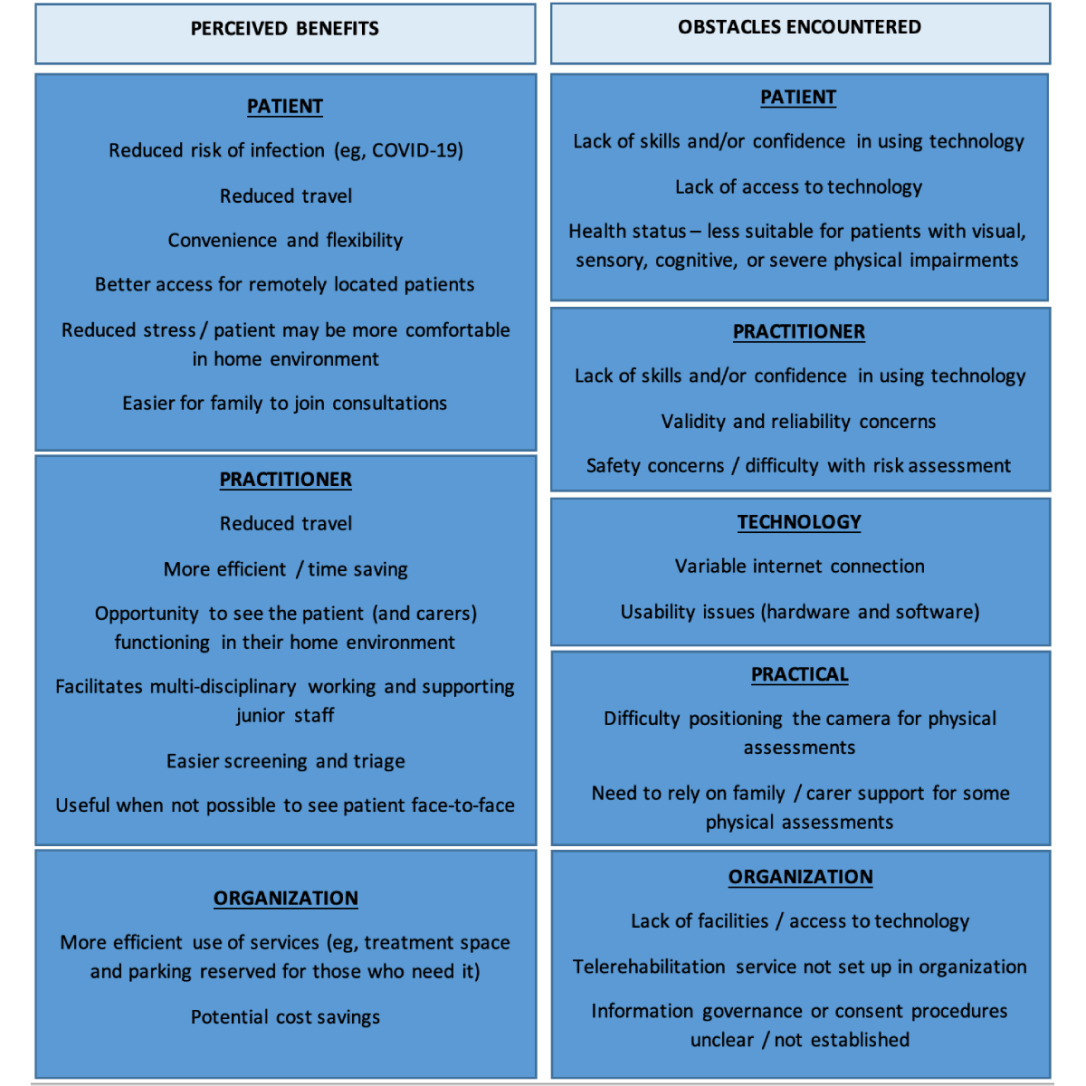
Benefits and obstacles of video-based consultations (as perceived by practitioners).

Respondents were asked to select the three most important benefits of undertaking video-based consultations (Figure 1). The three most frequently selected benefits were patient-related, including reduced risk of infection (161/207, 78%), reduced patient travel (120/207, 58%), and convenience and flexibility of the appointment for patients (79/207, 38%). In open responses, reduced travel and improved flexibility were deemed particularly beneficial for those with physical disabilities and fatigue. A range of additional benefits were perceived for practitioners and organizations, including efficiency, facilitating multidisciplinary working, and cost savings (Figure 1).

Obstacles encountered by practitioners in relation to video-based consultations were grouped into five categories: technology related, practical, patient related, practitioner related, and organizational (Figure 1). Technology-related issues had been experienced by 180 of 207 (87%) respondents. These included poor internet connections and usability issues (eg, performance, responsiveness, and incompatibility of hardware and software). Practical issues, including difficulty positioning the camera for physical assessments, had been experienced by approximately 71% (146/207) of respondents. Patient-related issues included lack of skills or confidence in using technology (experienced by 149/207, 72% of respondents) and lack of access to technology (reported by 151/207, 73%). The patient’s health status was also a frequently encountered obstacle. Around 56% (116/207) of respondents perceived telerehabilitation as less suitable for people with visual, sensory, or cognitive impairments, and 46% (95/207) reported severe physical impairment as an obstacle to a successful remote consultation. Practitioner-related obstacles included a perceived lack of skills or confidence in using technology (reported by 15/207, 15%), concerns surrounding the validity and reliability of video-based assessments (71/207, 34%), and safety concerns or difficulties conducting a risk assessment remotely (49/207, 24%). Organizational and governance obstacles were encountered by 21 of 207 (10%) respondents (eg, organizations recommending face-to-face consultations or prohibiting the use of certain technologies).

For the respondents who had not used video-based consultations (40/247, 16%), the reasons given were closely related to the obstacles experienced by the practitioners already using telerehabilitation. However, more emphasis was placed on organizational factors. These included unavailability of the required hardware or software within the organization and telerehabilitation services and protocols not having been set up yet.

Practitioners reported that technical and practical obstacles were most overcome by support from family members or carers. This support included helping to use the technology, holding and positioning the camera during physical assessments, ensuring the environment is safe and free of obstacles, providing physical support (eg, when assessing balance), and clarifying the practitioner’s instructions.

#### Physical Outcomes Assessed Remotely

Half (103/207, 50%) of the respondents had used video-based consultations to assess physical impairments (aspects such as strength and joint range as distinct from physical function). The categories of physical impairments most frequently assessed remotely were generalized (gross) and specific (individual joints) range of movement, posture, and balance (Table 3). Physical function had been assessed remotely by 107 of 207 (52%) practitioners, including standardized tests such as the Five Times Sit-To-Stand [29] and Timed Up and Go [30] tests, and nonstandardized assessments such as observing gait. Patient-reported measures had been used remotely by 121 of 207 (59%) practitioners, with activities of daily living and pain assessed most frequently (Table 3).

Practitioners reported a number of specific concerns in relation to the validity, reliability, and safety of clinician-rated physical assessments carried out remotely. These were grouped into five key themes (Table 4). The most frequently reported concern was a lack of confidence in applying physical measures remotely when they had not been designed for remote use:

I am concerned that we are all using assessment techniques which lack known reliability and validity if conducted in a context different to that in which they are supposed to be used (i.e. using them remotely rather than face-to-face).

Physiotherapist, Musculoskeletal

Additional concerns included physical examination restrictions preventing a hands-on approach (assessing muscle tone, strength, sensation, etc), communication difficulties, technology issues, and concerns about patient safety. Safety was a particular concern in cases where the patient was alone. Although only 10 of 207 (5%) respondents had experienced a safety incident (eg, a fall or near miss) while conducting remote physical assessments, practitioners reported being more risk averse compared with face-to-face consultations. This led to a reduction in the number of assessments carried out, and some avoided these assessments altogether:

Among some colleagues I noticed a perceived fear regarding the safety of remote interventions and this dominated so they were reluctant to consider any remote interventions or even reviews.

Physiotherapist, Neurology

Most practitioners accepted the validity and reliability of patient-reported outcomes, but a small number reported concerns when using these measures remotely. Concerns included accuracy and reliability of self-reported measures and the potential influence of family members:

I don’t have any evidence of reliability of taking patient-reported outcome measures by video or how much pressure the parents are using.

Physiotherapist, Pediatrics

**Table 3:** Physical impairments and patient-reported outcomes assessed remotely.

		Respondents, n (%)^a^
**Clinician-rated measures of physical impairment (n=103)^b^**		
	Range of motion: generalized (eg, gross lower limb movement)	84 (82)
	Posture	66 (64)
	Range of motion: specific (individual joints)	63 (61)
	Balance	60 (58)
	Dexterity	34 (33)
	Muscle strength	40 (39)
	Speech	12 (12)
	Swallowing	5 (5)
	Respiratory	5 (5)
	Other^c^	19 (18)
**Patient-reported outcome (n=121)^d^**		
	Activities of daily living	79 (65)
	Pain	78 (65)
	Fatigue	59 (49)
	Quality of life	55 (46)
	Psychosocial	40 (33)
	Cognitive	22 (18)
	Other^e^	31 (26)

^a^Some respondents reported assessing multiple impairments or outcomes; therefore, the percentages do not total 100.

^b^Respondents were asked “When undertaking video consultations, which of the following physical impairments do you measure remotely?”

^c^Other impairments included muscle tone, tremor, reflexes (including vestibulo-ocular), bradykinesia, facial palsy, skin disorders and scars, oedema, and congenital impairments (eg, arthrogryposis, radial longitudinal deficiency, and syndactyly).

^d^Respondents were asked “Which of the following do you assess remotely using self-report?”

^e^Other patient-reported outcomes assessed remotely included movement, general health status, and sensory function.

**Table 4:** Concerns of practitioners regarding the validity and reliability of remote physical assessments.

Theme	Description	Exemplar quote
Lack of confidence in measures used remotely	Practitioners expressed distrust and skepticism in the accuracy and reliability of the measures they used, as they felt they were not designed to be used remotely. This led to uncertainty about the effectiveness of interventions. Practitioners described taking outcome measures with a “spoonful of salt” and used them as a general indication of health, rather than to evaluate change. This theme was the most frequent concern out of all responses.	“We are having to use observations which have unknown reliability and validity when used remotely.” (Physiotherapist, Musculoskeletal)
Physical examination restrictions	Physical examinations were reported as being considerably restricted when working remotely. Examples of problems were a limited view of the patient due to the camera angle, not feeling the movements of the patient, and difficulty gaining a valid assessment of mobility. This theme was the second most frequent concern.	“It’s easy to miss things over video. You can’t always see all the movement.” (Occupational Therapist, Generic)
Patient safety concerns	Practitioners were concerned for the patient’s safety when engaging in physical assessments. As they were not physically present, they felt that they were not in control of the patient’s environment, and therefore unable to minimize the risk of falls or other safety incidents.	“Safety can be a real concern. If the person is at risk of falls then you need a carer by them or otherwise I don't undertake the test.” (Physiotherapist, Neurology)
Communication issues	Communication issues between the patient and practitioner related to information clarity and ensuring the patient understood instructions during assessments. Practitioners expressed that the lack of nonverbal cues and body language could hinder rapport building. Some concerns also revolved around distractions in the patient’s home environment.	“There is less rapport [online] so I feel that the client is less likely to reliably report how they are managing [their condition].” (Occupational Therapist, Neurology)
Technology issues	Practitioners reported that technical issues including hardware and internet connections impacted on their ability to carry out physical assessments. Poor quality of video and time lags reduced visual acuity and ability to discern subtle changes in movement.	“It is sometimes difficult to visually pick up all aspects due to poor internet connection.” (Physiotherapist, Pediatrics)

### Self-Perceived Confidence and Competence

**Figure 2:**
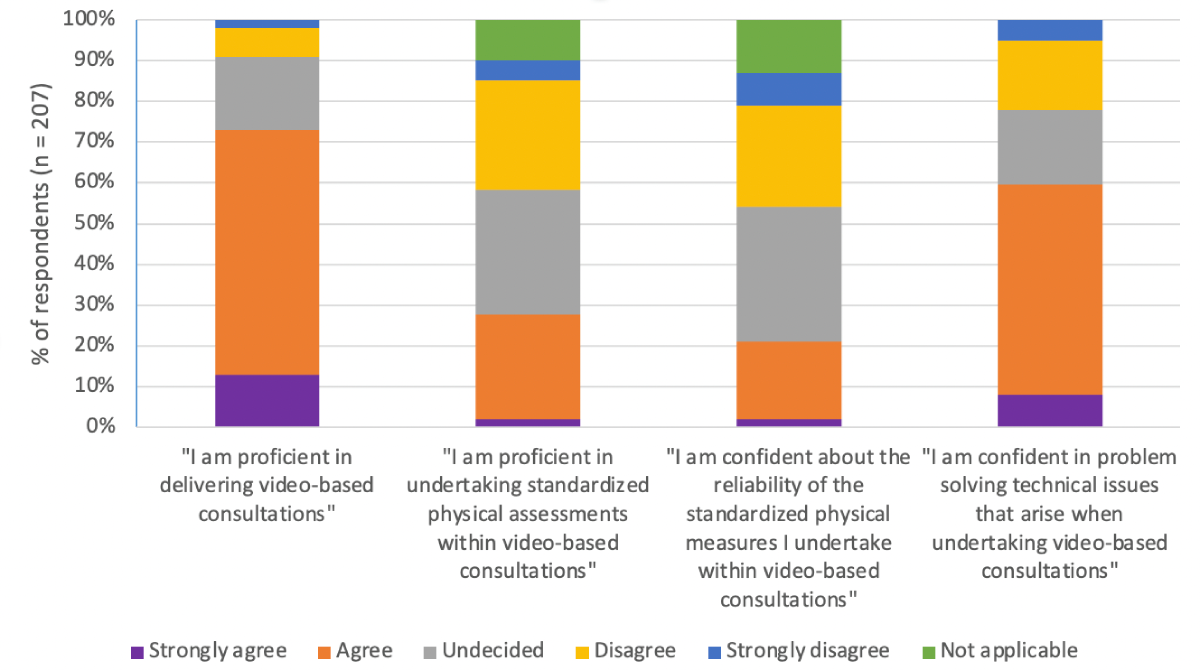
Self-perceived competence and confidence in carrying out video-based consultations (n=207).

Practitioners who had carried out video-based consultations were asked to rate their self-perceived confidence and competence (Figure 2). Although most (150/207, 72%) respondents reported that they felt they were proficient in delivering video-based consultations, fewer felt proficient in undertaking physical assessments using this method. Of the 187 practitioners who had used standardized clinician-rated physical assessments within video-based consultations, 57 (30%) reported feeling proficient in conducting these assessments. Of the 207 practitioners, 123 (59%) felt confident in dealing with technical issues.

### Knowledge and Training Needs

Sources of information used by respondents regarding the use of telerehabilitation are shown in Table 5. The most frequent source of knowledge was informal sharing of information with colleagues (reported by 190/247, 77% of respondents). Almost half of the respondents (118/247, 48%) had referred to their organization’s standard operating procedure or guidance. Practitioners had accessed a range of online sources including webinars, social media, and blogs.

Only 46 (19%) practitioners had received formal training in telerehabilitation or video consultations; this was most frequently delivered in a virtual classroom. Many respondents reported having learned quickly through experience and recognized the need for improved guidance and training:

We are expected to provide telerehabilitation without guidance or training – we are feeling our way in the dark.

Physiotherapist, Neurology

We need explicit guidance about what should and what should not be expected from a video consultation.

Consultant, Neurology

It would be good to have guidance for an approach that works that can be adopted by a whole team. Everyone is making it up as they go along, so even within a service there is no consistency.

Physiotherapist, Neurology

Respondents desired training to be flexible and not too time-consuming to fit it around their work commitments. The majority stated that they would prefer a virtual classroom or a blended approach with facilitated and self-directed learning. Regardless of the preferred training format, respondents wanted opportunities for interaction and discussion with peers; this was seen as important to enable sharing of experiences and ways to overcome challenges.

There was a perception that training in telerehabilitation should be available for staff at all levels but may be particularly important for students and junior staff with less clinical experience. For example:

Most experienced clinicians have fully adapted to remote consultation and we depend on our experience, whereas students or new clinicians have no experience so remote appointments for them will be a little different to a simulation or text, they will lack the essential full sensory experience of a real patient.

Physiotherapist, Musculoskeletal

Respondents desired guidance on the following subjects:

- Physical measures and assessment tools that are suitable for remote use
- Governance including confidentiality and consent
- Guidance and support on different digital platforms (eg, Microsoft Teams)
- Examples of when to use video-based consultations or other methods (ie, telephone and face-to-face)
- Signposting to digital skills training for patients/service users and practitioners

**Table 5:** Sources of information used by practitioners in relation to video assessments or consultations (N=247).^a^

Source of information	Respondents, n (%)
Informally sharing information with colleagues	190 (77)
Own organization’s standard operating procedure/guidance	118 (48)
Published guidelines (eg, NHS^b^ Digital or professional guidelines)	76 (31)
Virtual working groups (eg, professional forums or special interest groups)	56 (23)
Social media (eg, Facebook or Twitter)	51 (21)
Journal articles (including Cochrane reviews)	42 (17)
YouTube videos	39 (16)
Webinars	8 (3)
Other online sources (eg, web searches, blogs, or help guides for video consultation platforms)	36 (15)

^a^Respondents were asked “Have you used any of the following sources of information relating to conducting video assessments or consultations? Please choose all that apply.”

^b^NHS: National Health Service.

### Best Practice and Recommendations

Survey respondents shared examples of successful practice, how they had overcome obstacles, and recommendations for telerehabilitation with people with physical disabilities and movement impairment. This included top tips for carrying out video-based consultations, outcome assessment measures and tools that have been successfully used remotely, and recommendations for working with specific groups (eg, people with cognitive or communication difficulties or patients recovering from COVID-19). The key recommendations are presented in Textbox 1.

**Recommendations of survey respondents in carrying out telerehabilitation with people with physical disabilities and movement impairment.**

- Support from family members and carers is crucial; they can provide physical assistance during assessments or help with using technology.
- Clear communication between the practitioner and patient is even more important in remote consultations and assessments (eg, give clear instructions, do not rush, and use summaries and repeating back).
- Prepare as much as you can in advance; for example, send the patient resources that can be referred to during the consultation, familiarize yourself with the technology, and plan the structure of the consultation.
- At least the same amount of time should be allocated for remote physical assessments as face-to-face.
- Telephone triage is a valuable tool for assessing background, medical, and medication history, and deciding on the best method for follow-up treatment and management.
- Make use of patient-reported outcome measures as much as possible (eg, questionnaires for pain and quality of life).
- Do not try to do too much; focus on one or two key physical outcomes in a single session.
- For people recovering from COVID-19, try to do the consultation at a time of day when they are less fatigued, and keep the session short.
- Keep safety at the forefront and use your clinical judgement but try not to be too risk averse in physical assessments; remember the patient is already functioning in their own home.

## Discussion

### Principal Results and Comparison With Prior Work

In this survey of UK rehabilitation practitioners in health and social care, we found that the use of telerehabilitation for people with physical disabilities and movement impairment had increased rapidly since the COVID-19 pandemic. Practitioners generally viewed telerehabilitation positively and recognized many advantages for patients, including reduced risk of infection, increased flexibility, and reduced burden of travel for those with physical disabilities and fatigue. Video- and telephone-based consultations were perceived as a useful adjunct to, rather than a replacement for, face-to-face care. They were not felt to be appropriate for every individual or case; for example, remote consultations may be less suitable for manual therapy and people with severe cognitive, sensory, or physical impairments. These findings reflect those of other studies, where telerehabilitation has been reported as both feasible and acceptable to practitioners and health service users as part of the wider package of care [10,31-33].

The majority of existing surveys have focused on the overall experience of video consultations for practitioners and service users, including perceived acceptability, satisfaction, and communication [10,13,14,33-35]. This survey extends the scope of this research by exploring the physical and movement-oriented aspect of remote consultations and assessments. The categories of physical impairments most frequently assessed via video-based consultations were generalized (gross) and specific (individual joints) range of movement, posture, and balance. Practitioners had used patient-reported outcome measures more frequently than standardized clinician-rated tests.

A number of obstacles were identified in relation to carrying out video-based consultations with people with physical disabilities and movement impairment. The obstacles experienced by practitioners who were using video-based consultations were closely related to the reasons stated by those who had never used this method. Obstacles were grouped into five categories: technological (eg, poor internet connection or usability issues), practical (eg, difficulty positioning the camera), patient related (eg, lack of skills and access to technology), practitioner related (eg, validity and reliability concerns), and organizational (eg, lack of facilities or protocols not established for telerehabilitation). This complements the findings of existing studies; for example, Bower and colleagues [36] classed barriers to clinicians’ use of technology in neurorehabilitation into factors related to the technology itself, its users, and the organizational context [36]. Practical issues including difficulties with camera angles and limited fields of view have been recognized by other telehealth researchers [37,38]. The use of novel technologies (eg, wide-angle webcams and robotic movement tracking devices) that can improve the field of view and aid remote assessments of movement offers one potential avenue for exploration in research and practice [39].

Many of the respondents expressed concerns regarding the validity, reliability, and safety of physical assessments completed remotely. The largest concern related to the application of physical measures remotely when they had not been designed for remote use. Although there is some evidence for the validity and reliability of specific physical outcomes assessed remotely, such as a systematic review by Mani and colleagues [40], this knowledge needs to be built on and made available to practitioners and used in practice. A few respondents expressed concerns regarding the remote use of patient-reported outcomes, including lower accuracy and reliability when completed remotely, and the potential influence of family members. This is also worthy of further research.

In our survey, physical examination restrictions and the prevention of hands-on therapy were also a concern of practitioners; this concern has previously been reported by both practitioners and patients undertaking remote physical therapy [37,38,41]. Safety concerns caused practitioners to be more risk averse (and in some cases avoidant) when carrying out assessments via video or telephone. Although most patients will be seen by alternative means (particularly as COVID-19 restrictions are easing), there is a possibility that, for some, this will lead to delays in diagnosis or treatment. Understanding the safety risks associated with remote physical assessments, and how risk averseness may impact on the type and quality of rehabilitation offered, are important issues for exploration in future research. Practitioners should carry out a thorough risk assessment, in which the risks and benefits of different actions (performing the physical assessment remotely, seeing the patient face-to-face, or taking no action) are carefully considered.

Technical and practical support from family members and carers was reported by respondents as a major facilitator that helped to overcome obstacles and alleviate safety concerns. This reflects the findings of a recent case study, where the success of telerehabilitation for people with dementia during the COVID-19 pandemic was dependent on technical and physical support from caregivers [42]. In our survey, practitioners were less likely to carry out remote physical assessments with patients they deemed vulnerable (eg, at risk of falling) if they lacked the support of a family member or carer. In light of these findings, future research should explore the feasibility, safety, and practicalities of carrying out effective and safe telephone and video-based consultations with people who live alone or do not have a carer present.

A major contribution of this study is the exploration and identification of training needs of practitioners in relation to telerehabilitation. Only around one in five of the practitioners in our survey had completed formal training in telerehabilitation or video consultations; this closely matches the findings of an Australian survey of allied health clinicians delivering telehealth for musculoskeletal conditions, where only 21% had received training [37]. In the Australian survey, there was a general feeling of lacking adequate training and support [37] similar to our survey where practitioners reported “feeling their way in the dark.” We found that practitioners had quickly adapted their ways of working through the COVID-19 pandemic and primarily learned through experience, relying on informal sources of knowledge such as sharing information with colleagues and social media. Although learning through experience is an important part of clinical practice [34,43], there is a need for improved resources, guidance, upskilling, and training to support this [37,44,45]. Our survey confirmed this, identified specific training needs and preferences, and captured recommendations and tips from practitioners working in telerehabilitation.

Regarding specific training needs, most respondents wanted training to take place in a virtual classroom or to involve a blended approach with facilitated and self-directed learning, with opportunities for peer discussion. In particular, a need for training in conducting remote physical assessments was identified. Practitioners felt less confident and competent in delivering this aspect of care remotely compared with subjective assessments and information giving. Practitioners desired specific guidance on physical assessment tools suitable for remote use, when to use video-based consultations or alternative methods, governance issues, digital platforms, and signposting to digital skills training for themselves and their patients.

A strength of the survey is the capture of both quantitative and qualitative information on a range of aspects related to telerehabilitation. This detailed information is currently being triangulated with the findings of our literature review and service evaluation to produce a practical tool kit of resources and a training package to support practitioners in the remote rehabilitation of people with physical disabilities and movement impairment [46].

### Clinical and Policy Implications

Based on the findings of this survey, there are three key recommendations for clinical practice and policy:

1. Education, training, and upskilling of practitioners: Training should include not only technical skills but also practical and communication skills in remote consultations, and safety, validity, and reliability of remote physical assessments. Supporting staff in health and social care should also be trained in the aspects that are relevant to them (eg, information governance and consent in remote consultations).
2. Provision of access to the necessary equipment, resources, and suitable environments for telerehabilitation: As well as hardware and software, equipment and resources may include the use of novel technologies (eg, robotic movement tracking devices) to help to overcome some of the practical obstacles encountered in movement assessments. Practitioners should have access to private, spacious, quiet rooms with good lighting.
3. Implementation and use of standardized protocols for telerehabilitation: Standardized guidance on aspects of telerehabilitation, such as governance, safety, and consent should be made available. Some tailoring will be necessary based on the needs of organizations and patients, but the adoption of such protocols will improve communication and consistency of care within and between health and social care services.

### Limitations

Some limitations should be considered, including the representativeness of the sample. Recruitment relied on opportunity and snowball sampling rather than random selection. These sampling methods were used for practical reasons, as it was necessary to capture data from practitioners as efficiently as possible to inform the rapid development of the tool kit. The high proportion of female respondents (82%) may be questioned, but this is representative of the health and social care workforce in the United Kingdom, as 77% of NHS staff [47] and 82% of adult social care staff [48] are female. To ensure the views of rehabilitation practitioners across a wide range of sites and work settings were represented, invitations were sent to a variety of national networks. As this was a UK sample, the international relevance of the findings may be questioned. The decision to select UK-based practitioners was a pragmatic one, as the tool kit will include specific guidance on aspects such as information governance and digital platforms used in the United Kingdom. Nevertheless, many of the issues identified are likely to be of relevance to other countries, as suggested by our scoping review [17].

It should be recognized that the online nature of the survey might have biased recruitment toward those who are more comfortable with digital technology and online working. In addition, the survey was cross-sectional, and the views and training needs of practitioners may change over time. Future surveys and qualitative studies should explore how experiences, attitudes, and training needs evolve during and after the COVID-19 pandemic. Finally, future research should explore the impact of clinical experience on confidence and proficiency in delivering telerehabilitation.

### Conclusions

This survey provided a comprehensive understanding of the experiences and training needs of UK health and social care practitioners regarding the use of telerehabilitation for people with physical disabilities and movement impairment. Although practitioners have rapidly adopted remote ways of working and viewed telerehabilitation positively overall, there are technical, practical, and organizational obstacles to overcome to maximize the success of this approach. There is a clear need for improved guidance and training, particularly surrounding physical and movement-oriented assessments. The findings will be of interest to practitioners, service providers, researchers, and technology developers, and will have practical relevance through informing the rapid and timely development of a tool kit of resources and a training package for the current and future workforce.

## Appendix

Multimedia Appendix 1 Questionnaire.

## Abbreviations

CHERRIES: Checklist for Reporting Results of Internet E-Surveys
NHS: National Health Service
UKRI-MRC: UK Research and Innovation–Medical Research Council
