# Supplementary material for: Telerehabilitation for People With Physical Disabilities and Movement Impairment: A Survey of United Kingdom Practitioners
Source: JMIRx Med. 2022 Jan 3;3(1):e30516. doi: 10.2196/30516 (PMC10414449; doi:10.2196/30516)
Supplement: Multimedia Appendix 1 [file xmed_v3i1e30516_app1.pdf]

Telerehabilitation survey

Introduction and information

UNIVERSITY OF PLYMOUTH
Faculty of Health

Thank you for showing an interest in this project. Please read this information carefully before deciding whether or not to participate.

What is the aim of the project?

The survey is part of a telerehabilitation research project and service evaluation led by researchers from the School of Health Professions at the University of Plymouth, with collaborators from across NHS and Adult and Children's Social Care Services in the South West of England. The project is funded by the Medical Research Council.

The aim of the survey is to explore the experiences, knowledge and needs of health and social services practitioners relating to telerehabilitation (the delivery of rehabilitation services using telephone and web-based consultations). In this project, we are focusing on the remote assessment of patients or service users with physical disabilities and movement impairment, including those recovering from COVID-19. The findings will be used to inform the development of a toolkit of resources and a training package for the current and future workforce.

Am I eligible to take part in this survey?

If you have direct patient contact (even if this is only occasional for research or educational purposes) and work in the NHS, Social Services, independent private or charitable organisation sectors, then we are keen to hear your views. We are looking for practitioners, from a wide range of disciplines, who have and have not used video-based consultations or assessments for people with physical disabilities and movement impairment.

What will I be asked to do?

You will be asked to complete an online questionnaire. The questionnaire should take around 15 minutes to complete.

Completion of the questionnaire does not commit you to further engagement in the project, but at the end of the questionnaire you will be given the opportunity to leave your contact details to discuss your responses with a researcher in more detail (this is entirely optional). Your details will only be used for this purpose.

Can I change my mind and withdraw from the project?

You may withdraw from participation without any disadvantage to yourself of any kind. Please note that after the survey submission deadline has passed, due to responses being collated immediately, it will only be possible to remove your contact details from our database. You may request to remove your details by e-mailing: telerehab@plymouth.ac.uk.

What data or information will be collected and what use will be made of it?

What information is being collected?

We will collect information on your experiences of delivering telerehabilitation, your knowledge of telerehabilitation (e.g. where you get information from), training needs, and perceived competence and confidence in conducting video-based assessments. We will also collect some basic demographic information (your age, gender and occupation).

How will my data be handled?

Your individual answers will only be seen by the telerehabilitation research team. Data will be stored securely at the University of Plymouth for 10 years in line with University policy. The processing, handling and storage of data will be in accordance with the General Data Protection Regulation (GDPR) and Data Protection Act 2018. All responses will be anonymised for reporting and analysis.

Can I see a summary of the findings?

Yes – if you would like a summary of the findings of the survey (and the wider project) there will be an opportunity to leave your contact details at the end of the questionnaire.

Who should I contact if I have a complaint?

If you have any complaints about the way in which this study has been carried out please contact the research team in the first instance (telerehab@plymouth.ac.uk). If your complaint is not resolved please contact the Faculty of Health Research Ethics administrator: FOHEthics@plymouth.ac.uk.

How can I find out more about the project?

If you have any questions about the project or would like to find out about other ways you can be involved, please e-mail: telerehab@plymouth.ac.uk.

The research team:

Prof Jenny Freeman (Principal Investigator), Dr Sarah Buckingham, Dr Kim Sein, Prof Ray Jones, Prof Diane Playford, Prof Jonathan Marsden, Prof Bridie Kent, Prof Sara Demain, Dr Hilary Gunn, Dr Angie Logan, Krithika Anil, Barbara Scott.

NHS and Social Service Collaborators:

Dr Rachel Botell, Dr Thorsten Brandt, Emma Cork, Sian Goddard, Sandra Houston, Hazel Alexander, Lucy Obolensky, Rachel Rapson, Dr Chris Rollinson, Sarah Scrivener.

This project has been reviewed and approved by the University of Plymouth Faculty of Health Research Ethics and Integrity Committee.

Please click Next to move on to the consent page.

Consent

1. I have read the participant information and consent to take part in the survey. *
Required

- ◯ Yes
- ◯ No

Demographics

Please answer all of the questions as fully as possible based on your own experiences (rather than from an organisational perspective).

Note: Questions with asterisks * must be completed for the survey to move on to the next page.

2. Please enter your age in years: *Optional*

3. Gender - please choose only **one** of the following: ✱ *Required*

- Male
- Female
- Other
- Prefer not to say

4. Setting of service (please select the **one** that best describes your service): ✱ *Required*

4.a. If you selected Other, please specify:

5. Profession - please select **one** option: ✱ *Required*

5.a. If student, please specify:

5.b. If medic, please specify:

5.c. If you selected Other, please specify:

6. Clinical speciality - please choose only **one** of the following: ✱ *Required*

- Amputees
- Cardiology (including cardiac rehabilitation)
- Care of older people
- Community rehabilitation
- Falls
- Generic
- Intensive/critical care
- Learning disabilities
- Mental health (adults or children)
- Musculoskeletal/rheumatology
- Neurological (including stroke rehabilitation)
- Occupational health

- Oncology
- Pain management
- Palliative care
- Rapid Response
- Respiratory (including pulmonary rehabilitation)
- Sports and exercise
- Trauma and orthopaedics
- Women's/men's health
- Academic/education
- Research
- Other

6.a. If you selected Other, please specify:

7. Do you work mainly with children or adults? (Please choose only **one** option) ✱ *Required*

- Children/adolescents
- Adults

8. Location – please select **one** option: ✱ *Required*

8.a. If you selected Other, please specify:

9. Do you predominately deliver your service to patients/service users in…? (please choose only **one** of the following) ✱ *Required*

- An urban setting
- A rural setting
- Both

Experience of telerehabilitation 1

Use of video-based consultations

10. Have you ever used video-based consultations? ✱ *Required*

- ○ Yes
- ○ No

10.a. Please explain why you have never used video-based consultations:

Experience of telerehabilitation 2

Use of video-based consultations

11. Which of the following best describes your use of video-based consultations (including for rehabilitation or other purposes)? Please choose **one option in each row**.

	✱ *Required*		
	Not at all	Occasionally	Frequently
Used pre-COVID lockdown (prior to March 2020)	○	○	○
Used during COVID lockdown (March to June 2020)	○	○	○
Used post-COVID lockdown (July 2020 to present)	○	○	○

12. For which of the following purposes have you used video-based consultations? Please choose **all** that apply. ✱ *Required*

- ☐ Screening and triage
- ☐ Initial assessment
- ☐ Follow-up assessments
- ☐ Assess and/or review use of equipment
- ☐ Intervention delivery on an individual basis (e.g. goal-setting, exercise, education)
- ☐ Intervention delivery on a group basis (e.g. exercise class, educational class)
- ☐ Other

12.a. If you selected Other, please specify:

13. Within which of the following settings have you used video-based consultations? Please choose **all** that apply: ✱ *Required*

- ☐ My home
- ☐ My workplace
- ☐ Other

13.a. If you selected Other, please specify:

14. Do you have access to an IT or technical support team who can help with technological queries or problems arising in video-based consultations? Please choose only **one** option: ✱ *Required*

- ◯ Yes
- ◯ No
- ◯ I don't know

15. Have you used telerehabilitation with patients who have been diagnosed with COVID-19? Please choose only **one** of the following: ✱ *Required*

- ◯ Yes, telephone consultation
- ◯ Yes, video-based consultation

- Yes, telephone and video-based consultations
- No

Experience of telerehabilitation 3

Mode of delivery and digital platform(s)

16. Which digital platform(s) do you use for video consultations or assessments? Please choose **all** that apply. ✱ *Required*

- ☐ AccuRx
- ☐ AskmyGP
- ☐ Attend Anywhere
- ☐ CISCO Webex
- ☐ EMIS Health
- ☐ Facebook video calls/Facebook Portal
- ☐ FaceTime
- ☐ GoodSAM
- ☐ Google Meet
- ☐ Lifesize
- ☐ Microsoft Teams
- ☐ NHS Near Me
- ☐ OneConsultation
- ☐ Pexip
- ☐ Push Dr
- ☐ Referro
- ☐ Savience/HSL
- ☐ Skype
- ☐ Vimeo
- ☐ Visionable
- ☐ WhatsApp video calls
- ☐ XuperVisconn
- ☐ Zoom
- ☐ Other

16.a. If you selected Other, please specify:

17. Why were these particular platforms selected? Please choose **all** that apply. ✱
Required

- ☐ My preference
- ☐ Patient/service user's preference
- ☐ My organisation stipulates the use of these platforms
- ☐ Recommended by guidance
- ☐ Other

17.a. If you selected 'recommended by guidance', please specify the guidance you used:

17.b. If you selected Other, please specify:

18. Which other methods of contact have you used to support your video-based

assessment? Please choose **all** that apply. ✱ *Required*

- ☐ Telephone
- ☐ E-mail
- ☐ Text messaging
- ☐ App-based messaging (e.g. WhatsApp)
- ☐ Post
- ☐ Face-to-face
- ☐ Other
- ☐ None of the above

18.a. If you selected Other, please specify:

19. Have you used video recordings in remote consultations? Please choose **all** that apply: ✱ *Required*

- ☐ Yes, taken by the patient/service user and reviewed during the consultation
- ☐ Yes, taken by the patient/service user and reviewed after the consultation
- ☐ Yes, taken by me and reviewed during the consultation
- ☐ Yes, taken by me and reviewed after the consultation
- ☐ No

19.a. Please comment on how you use and store these videos:

Experience of telerehabilitation 4

Perceived benefits and obstacles

20. Which of the following do you perceive as the **three most important benefits** of video-based consultations for remote patient assessments? Please choose **three** options: ✱ *Required*

Please select exactly 3 answer(s).

- [ ] Good for out-of-hours
- [ ] More efficient/saves time for clinicians
- [ ] More information is available to me
- [ ] Reduces clinicians' travel
- [ ] Reduces patient/service user's travel
- [ ] Better access for remotely located patients/service users
- [ ] Better patient/service user and/or family engagement
- [ ] Less stressful for patients/service users and their families
- [ ] More convenient for the patient/service user
- [ ] Reduced risk of infection (e.g. COVID-19)
- [ ] Other

20.a. If you selected Other, please specify:

21. Which of the following issues or **obstacles** to conducting video-based consultations have you experienced? Please choose **all** that apply: ✱ *Required*

- [ ] Technology-related issues (e.g. poor internet connection, lack of facilities, usability issues)
- [ ] Practical issues (e.g. difficulty positioning camera for physical assessments)
- [ ] I lack the necessary skills and/or confidence in using technology
- [ ] The patient/service user does not have access to the necessary technology (e.g. computer, smartphone, broadband)
- [ ] The patient/service user lacks skills and/or confidence in using technology
- [ ] Less suitable for people with severe physical impairments (i.e. inhibiting the use of technology)
- [ ] Less suitable for people with visual, sensory or cognitive impairment
- [ ] Less suitable for people with anxiety
- [ ] Low patient/service user engagement
- [ ] Need to rely on family/carer engagement
- [ ] More difficult to build rapport with patient/service user
- [ ] Organisational or governance issues (e.g. my organisation recommends face-to-face consultations or prohibits the use of certain technologies)
- [ ] Issues with obtaining patient/service user's consent
- [ ] Safety concerns (e.g. risk of falls)
- [ ] Concerns about validity and/or reliability of remote assessments
- [ ] Privacy/confidentiality concerns
- [ ] Other
- [ ] None of the above

21.a. If you selected Other, please specify:

Experience of telerehabilitation 5

Measurements

22. Do you measure **physical impairments** during video-based consultations? ✱
Required

- Yes
- No

22.a. When undertaking video consultations, which of the following physical impairments do you measure remotely? Please choose **all** that apply:

- Balance
- Dexterity
- Muscle Strength
- Range of motion: generalised (e.g. gross lower limb movement)
- Range of motion: specific (individual joints)
- Respiratory
- Posture
- Speech
- Swallowing
- Other

22.a.i. If you selected Other, please specify:

22.b. What are your recommended **top three** standardised measures of physical

impairment that you have used successfully in your video consultations?

23. Do you measure **physical function** as part of video-based consultations? (e.g. sit-to-stand, walking) ✱ *Required*

- Yes
- No

23.a. What are your recommended **top three** standardised functional measures (e.g. five-repetition sit-to-stand, 10 metre walk test) that can be used in video consultations?

24. Do you use **self-report measures** as part of video-based consultations? ✱ *Required*

- Yes
- No

24.a. Which of the following do you assess using self-report? Please choose **all** that apply.

- [ ] Activities of Daily Living
- [ ] Cognitive

- [ ] Fatigue
- [ ] Pain
- [ ] Psychosocial (including depression/anxiety)
- [ ] Quality of Life
- [ ] Other

24.a.i. If you selected Other, please specify:

24.b. Please specify which self-report measures you use (e.g. EQ-5D, HADS):

25. Do you have any concerns regarding the validity or reliability of any of the measures you use for remote assessments? ✱ *Required*

- ○ Yes
- ○ No

25.a. Please give details of these concerns or how you have overcome them:

26. Have you experienced any safety incidents (e.g. falls) while conducting remote physical assessments? ✱ *Required*

- ◯ Yes
- ◯ No

26.a. Please give details:

27. Please state your **top tip** for carrying out video-based consultations where the focus is on assessing physical impairments and movement:

28. Do you have any recommendations or tips for carrying out video-based consultations **specifically for patients recovering from COVID-19**? ✱ *Required*

- ◯ Yes
- ◯ No

28.a. If yes, please describe here:

Perceived competence

29. Please rate the following statements: ✱ *Required*

Please don't select more than 1 answer(s) per row.

Please select at least 4 answer(s).

	Strongly agree	Agree	Undecided	Disagree	Strongly disagree	Not applicable
I am proficient in delivering video-based consultations.	☐	☐	☐	☐	☐	☐
I am proficient in undertaking standardised physical assessments within video-based consultations.	☐	☐	☐	☐	☐	☐
I am confident about the reliability of the standardised physical measures I undertake within video-based consultations.	☐	☐	☐	☐	☐	☐

I am confident in problem solving technical issues that arise when undertaking video-based consultations.	☐	☐	☐	☐	☐	☐

Knowledge and training

Sources of information

30. Have you used any of the following sources of information relating to conducting video assessments or consultations? Please choose **all** that apply: ✱ *Required*

Please select at least 1 answer(s).

- [ ] Informally sharing information with colleagues
- [ ] Journal articles (including Cochrane reviews)
- [ ] Published guidelines (e.g. NHS Digital, NHSX, NHS England, professional guidelines)
- [ ] Social media (e.g. Facebook, Twitter)
- [ ] Standardised operating procedure/guidance produced by my organisation
- [ ] Virtual working groups (e.g. professional forums, special interest groups)
- [ ] YouTube videos
- [ ] Other online sources (e.g. websites, blogs)
- [ ] Other
- [ ] None of the above

30.a. If you selected Other, please specify:

30.b. If you selected Published guidelines, please specify:

30.c. If you selected Virtual working groups, please specify:

30.d. If you selected Other online sources, please specify:

Training in telerehabilitation

31. Have you completed any formal training in telerehabilitation or video consultations? ✱ *Required*

- ○ Yes
- ○ No

31.a. How was this training delivered? Please choose **all** that apply:

- ☐ Face-to-face instructor-led course
- ☐ E-learning (no instructor)
- ☐ Virtual classroom (with a host/instructor)
- ☐ Other

31.a.i. If you selected Other, please specify:

Training needs

32. Which of the following types of information would you like to see included in an online telerehabilitation training toolkit for health and social care professionals? Please choose **all** that apply: ✱ *Required*

Please select at least 1 answer(s).

- ☐ Digital platforms/programs that may be used for video consultations or assessments
- ☐ Guidance on when to use video consultations versus other methods (e.g. face-to-face or telephone)
- ☐ Information on governance of video consultations (e.g. confidentiality, consent)
- ☐ Recommended remote measures/assessment tools for physical impairment, physical function and self-reported outcomes
- ☐ Safety information (e.g. environmental considerations when conducting remote assessments)
- ☐ Signposting to digital skills training for patients/service users
- ☐ Signposting to digital skills training for myself
- ☐ Specialist guides – such as assessment of children, older people, people with communication/cognitive difficulties
- ☐ Technical considerations (e.g. broadband speeds required for movement assessment)
- ☐ Other
- ☐ None of the above

32.a. If you selected Other, please specify:

32.b. Please specify the type of specialist guides that would be useful (e.g. children, older people):

33. Which of the following resources would you like to see included in an online telerehabilitation toolkit? Please choose **all** that apply: ✱ *Required*

Please select at least 1 answer(s).

- ☐ Case studies/examples of good practice
- ☐ Checklists for consultations
- ☐ Example script for carrying out remote consultations
- ☐ Frequently asked questions
- ☐ Library of resources
- ☐ Self-reflection tools to reflect on/evaluate own knowledge/skills
- ☐ Top tips
- ☐ Videos/tutorials
- ☐ Other
- ☐ None of the above

33.a. If you selected Other, please specify:

34. Of the following options, what type of training would you prefer? Please choose only **one** option: ✱ *Required*

- Face-to-face instructor-led course
- E-learning (no instructor)
- Virtual classroom (with a host/instructor)
- A blended approach/combination of these

34.a. Please specify:

35. Please add any further comments relating to information or training needs:

Final comments and contact information

36. If you have any further comments on any issue about telerehabilitation or anything within the survey please add these here:

37. Would you be willing to be contacted by the research team for either of the following? Please choose **all** that apply. ✱ *Required*

- ☐ Yes, I would be willing to discuss my survey responses in more detail
- ☐ Yes, I would like to be updated about the findings of this project
- ☐ No

37.a. Name:

37.b. E-mail address:

37.c. Telephone number:

37.c.i. Best days/times to call you:

Final page

Thank you very much for taking part in this survey. If you have any further questions about the project, please contact: telerehab@plymouth.ac.uk. If you wish to withdraw your responses before the survey submission deadline, or to request to remove your contact details from our database at any point in the future, please e-mail: telerehab@plymouth.ac.uk.

Key for selection options

4 - Setting of service (please select the one that best describes your service):

Primary care (e.g. GP surgeries, First Contact Practitioners)
Secondary care (e.g. hospital services, outpatient clinics, child development services)
Tertiary care (e.g. specialist hospitals)
Community health and/or social care
Residential social care
Private practice
Social enterprise
Academic institution
Charity
Other

5 - Profession - please select one option:

Physiotherapist
Occupational therapist
Speech and language therapist
Nurse
Medic
Student
Other

8 - Location – please select one option:

England
Wales
Scotland

Northern Ireland
Channel Islands
Isle of Man
Other
